# Detecting microvascular changes in the mouse spleen using optical computed tomography

**DOI:** 10.1016/j.mvr.2015.06.008

**Published:** 2015-09

**Authors:** Ciara M. McErlean, Jessica K.R. Boult, David J. Collins, Martin O. Leach, Simon P. Robinson, Simon J. Doran

**Affiliations:** a Cancer Research UK Cancer Imaging Centre, The Institute of Cancer Research, Sutton SM2 5NG, UK; b Royal Marsden NHS Foundation Trust, Sutton, Surrey SM2 5PT, UK; c University of Surrey, Guildford, Surrey GU2 7XH, UK

**Keywords:** BABB, benzyl benzoate:benzyl alcohol, BV, blood vessel, CMOS, complementary metal-oxide semiconductor, CT, computed tomography, FOV, field-of-view, GLCM, grey-level co-occurrence matrix, LN, lymph node, LV, lymph vessel, MZ, marginal zone, OPT, Optical Projection Tomography, PBS, phosphate-buffered saline, RBC, red blood cell, ROI, region-of-interest, RP, red pulp, SEM, scanning electron microscope, SNR, signal-to-noise ratio, TEM, transmission electron microscope, VDA, vascular disrupting agent, WP, white pulp, Toxicity, ZD6126, Optical computed tomography, Spleen microvasculature, Textural analysis

## Abstract

Methods of monitoring drug toxicity in off-target organs are very important in the development of effective and safe drugs. Standard 2-D techniques, such as histology, are prone to sampling errors and can miss important information. We demonstrate a novel application of optical computed tomography (CT) imaging to quantitatively assess, in 3-D, the response of adult murine spleen to off-target drug toxicity induced by treatment with the vascular disrupting agent ZD6126. Reconstructed images from optical CT scans sensitive to haemoglobin absorption reveal detailed, high-contrast 3-D maps of splenic structure and microvasculature. A significant difference in total splenic volume was found between vehicle and ZD6126-treated cohorts, with mean volumes of 61 ± 3 mm^3^ and 44 ± 3 mm^3^ respectively (both n = 3, p = 0.05). Textural statistics for each sample were calculated using grey-level co-occurrence matrices (GLCMs). Standard 2-D GLCM analysis was found to be slice-dependent while 3-D GLCM contrast and homogeneity analysis resulted in separation of the vehicle and ZD6126-treated cohorts over a range of length scales.

## Highlights

• Murine spleen imaged in 3-D using optical computed tomography
• Endogenous haemoglobin-sensitive contrast revealed detailed internal structure.
• Toxicity effect of treatment with vascular disrupting agent ZD6126 characterised
• Significant ZD6126 treatment-induced shrinkage of 28% of total spleen volume found
• Microvascular ZD6126 treatment-induced changes characterised via textural analysis

## Graphical abstract

**Figure:**
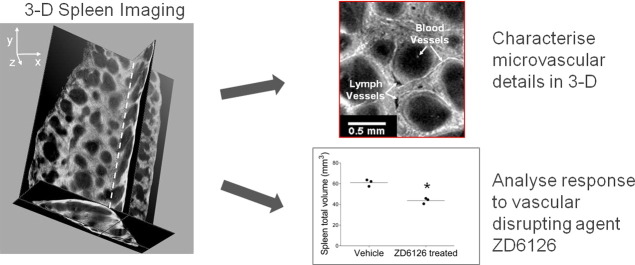

## Introduction

Toxicity is an important consideration in drug development and off-target effects are common, with potentially significant adverse side-effects. Methods that provide informative, accurate and rapid assessment of drug-induced toxicity to major target organs, such as the liver, kidneys and spleen, can either positively impact and accelerate the development of promising new agents, or force early closure of a programme that ultimately will not deliver a safe drug. They also inform design of subsequent clinical trials, ensuring that imaging protocols are optimised to include potentially critical normal tissues.

In the treatment of cancer, a number of agents designed to specifically target the tumour vasculature have been and continue to be developed. Given the hypervascular nature of the spleen, its essential role as a blood filter and the unique presence of acute endothelial contractility (Ragan et al., 1988), the effects of these vascular targeting agents on splenic perfusion are also commonly assessed. Typically, this has been achieved pre-clinically through the use of histological markers of perfused vasculature or tissue uptake of radiolabelled blood flow tracers (Cullis et al., 2006; Horsman and Murata, 2003). Splenic perfusion has also been assessed as part of clinical trials of vascular targeting agents by incorporating functional imaging, where the spleen is included in the imaging field-of-view (FOV) (Anderson et al., 2003; Evelhoch et al., 2004).

The spleen has a complex 3-D structure made up of several distinct tissue types. Extensive interconnections between red pulp, white pulp and intermediary marginal zones are integral to the organ's function (Groom, 1987). Traditional 2-D histology methods are not well suited for the examination of these 3-D features and they are often subject to sampling error due to the limitations of the physical sectioning techniques used. In addition, it is time-consuming to match information between adjacent slices, which may be distorted by the histological preparation process. A number of imaging approaches have been applied to the spleen, including microvascular-corrosion casting, scanning and transmission electron microscopy (SEM, TEM), and *in vivo* microscopy (Groom, 1987). These can have excellent resolution, but are often complex to perform and expensive.

Optical computed tomography (CT) is an emerging imaging modality. Although initially introduced in 1996 to solve problems in the physical sciences (Gore et al., 1996; Winfree et al., 1996), the first micro-imaging measurements on tissue were presented somewhat later, in 2002, using the Optical Projection Tomography (OPT) variant of the scanner geometry (Sharpe et al., 2002). This technique has subsequently been applied to the imaging of whole rodent organs and embryos in a variety of contexts (Oldham et al., 2007; Sharpe, 2003). The FOV size sits in the “imaging gap” between confocal microscopy and magnetic resonance imaging (MRI), ranging from a single cell to several centimetres, with resolution generally proportional to the FOV.

Prior to optical CT imaging, tissue samples must, in general, undergo a process of optical clearing to render them transparent at optical wavelengths (Oldham et al., 2008; Zhu et al., 2013). Optical CT images can be acquired in absorption or fluorescence modes, and the contrast observed can be either *endogenous* (related to the remnant optical absorption of tissue after the clearing process, or the tissue autofluorescence), or *exogenous* in the form of optical stains which are well characterised in the field of histopathology. Previously OPT has been used to probe developing spleens in embryos (Asayesh et al., 2006; Hecksher-Sørensen et al., 2004), but the technique has not been applied to adult mouse spleen in which the 3-D features are fully developed.

In this study, the ability of endogenous optical CT contrast to detect and to assess quantitatively the microstructure of normal adult murine spleen was investigated. In addition, structural changes were also characterised in spleens excised from mice following treatment with the vascular disrupting agent (VDA) ZD6126. A previous 2-D assessment, using fluorescence microscopy of Hoechst 33342 uptake (Cullis et al., 2006), demonstrated a significant reduction in splenic perfusion with this agent, making it a highly relevant case study.

## Materials and methods

### Tissue collection

ZD6126 (N-acetylcolchinol-*O*-phosphate, Angiogene Pharmaceuticals Ltd.) was formulated in 20% of 5% sodium carbonate and 80% phosphate-buffered saline (PBS).

All *in vivo* experiments were performed in accordance with the local ethical review panel, the UK Home Office Animals (Scientific Procedures) Act 1986, the United Kingdom National Cancer Research Institute guidelines for the welfare of animals in cancer research (Workman et al., 2010) and the ARRIVE (animal research: reporting in vivo experiments) guidelines (Kilkenny et al., 2010). Six-week-old female Balb/c mice were randomised to be treated with either vehicle alone (n = 3) or 200 mg/kg ZD6126 i.p. (n = 3). The small sample size was chosen to minimise animal use in this initial proof-of-concept study. No adverse effects were observed in any mice and after 24 h the mice were killed by cervical dislocation and the spleens excised and placed in 70% ethanol in PBS overnight at 4 °C.

### Optical CT imaging

Spleens were first embedded in 0.75% agarose (Sigma-Aldrich, Gillingham, UK) and kept in 70% ethanol in PBS overnight at room temperature. The samples were then dehydrated with three washes of 100% ethanol over three days. Optical clearing was achieved with a graded series of ethanol and 1:2 benzyl benzoate:benzyl alcohol (BABB) solutions (Sigma-Aldrich, Gillingham, UK), with washes of 30% and 70% 1:2 BABB in ethanol, each for one day, followed by two washes of 100% 1:2 BABB over a one week period.

**Fig. 1:**
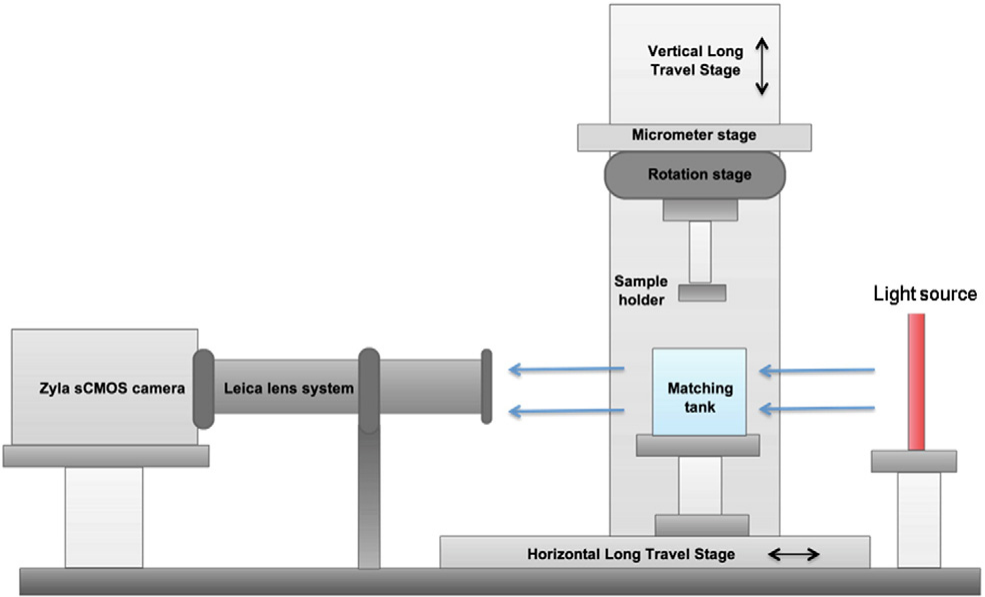
Diagram of the optical CT system used for imaging optically cleared spleen samples. Light passing through the sample was focused by a microscope lens system onto a camera chip and recorded in a ‘projection’ image for each rotation angle. See Doran et al. (2013) for details of individual components.

Imaging was performed using an in-house optical CT system, shown in Fig. 1, which was previously developed and well characterised for microbeam radiotherapy applications (Doran et al., 2013). Optical CT image reconstruction is similar to X-ray CT in that a series of ‘projection’ images are acquired, recording photon attenuation at different angles. Each sample was suspended from a sample holder and rotated 180° from above in a matching tank containing 1:2 BABB fluid, which has the same refractive index as the optically cleared samples. In order to achieve high resolution, a microscope zoom lens (Z16 APO zoom system, Leica Microsystems GmbH, Wetzlar, Germany) was used to focus each projection image onto a complementary metal-oxide semiconductor (CMOS) camera (Zyla sCMOS, Andor Technology PLC, Belfast, UK). Filtered backprojection was used to reconstruct axial slices through each sample from the many 2-D projection images.

Two datasets were acquired for each optically cleared spleen, each based on a raw dataset of 1000 projection images of 512 × 512 pixels, acquired over 180° rotation and reconstructed to a 512^3^-voxel volume. Dataset 1 had a FOV of (13.3 mm)^3^ and isotropic voxels of size (26 μm)^3^, while Dataset 2 had a FOV of (5.3 mm)^3^ and isotropic voxels of size (10.4 μm)^3^.

The first scan encompassed the entire spleen height, enabling accurate total volume measurements. The second scan had a reduced FOV to enable acquisition of higher resolution data over a limited height, providing more detailed images of the internal tissue structure. Dataset 1 was acquired using a wavelength of 630 nm (Flat-panel LEDR, Phlox, Aix-en-Provence, France). For increased contrast between the red and white pulp regions, a wavelength of 430 nm was chosen for Dataset 2 (SugarCUBE™ LED Illuminator, Nathaniel Group Inc. Vergennes, VT, USA and 430/10 nm bandpass filter, Thorlabs Inc., Newton, NJ, USA), which is near the peak absorption wavelength of deoxyhaemoglobin and also strongly absorbed by oxyhaemoglobin.

### Image analysis

Volume measurements were carried out by segmenting spleen from background in each slice and counting the total number of spleen voxels. Segmentation was done in OsiriX (Rosset et al., 2004). For each sample, images were imported as a stack of image files in JPEG format and the spleen was outlined manually every 20 axial slices. Using the ‘Generate Missing ROIs’ tool in OsiriX, outlines were interpolated for the other slices. Each slice was visually inspected to ensure accuracy with some minor adjustments made where necessary. The total volume of the outlined regions was calculated using OsiriX given the known voxel volume of (26 μm)^3^. This method is advantageous over threshold-based segmentation as highly attenuating artefacts were excluded. The volume results for each cohort were tested for significant difference using a non-parametric one-tailed Mann–Whitney U test at the 95% confidence limit.

**Fig. 2:**
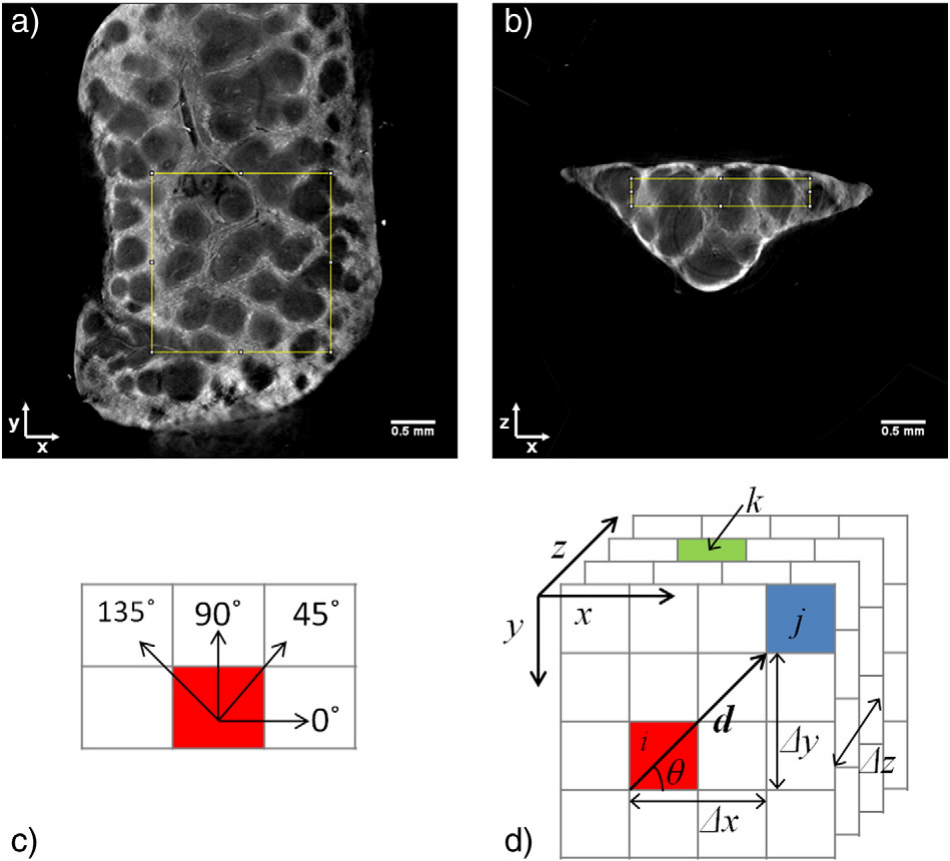
a. and b. Orthogonal cross-section slices of a reconstructed ZD6126-treated spleen image volume from Dataset 2 with FOV (5.3 mm)^3^. The yellow boxes indicate the position of the region-of-interest (ROI) (size 200 × 200 × 30 pixels) chosen for textural feature analysis. ROIs for the other samples were chosen to be in similar positions with respect to spleen boundaries. c. Representation of the four angular pixel directions used in grey level co-occurrence matrix (GLCM) calculations. For each pixel displacement, *d*, GLCMs were calculated in each angular direction and averaged to give one GLCM per displacement distance. d. Demonstration of the relative positions of three reference pixels used in 3-D GLCM calculations for the case of *d* = 2 and *θ* = 45°. For 2-D GLCM calculations only pixels *i* and *j* were compared. 3-D GLCM analysis incorporates depth information by including a third reference pixel, *k*, in the *z* direction. Pixel position changes Δ*x*, Δ*y* and Δ*z* can be calculated from Eq. (1).

Quantitative analysis of internal structures was performed by comparing various textural statistics between samples. Due to the irregular size and shapes of the spleens, regions-of-interest (ROIs) of size 200 × 200 × 30 pixels in similar positions were analysed for each sample, as shown in Fig. 2.

The grey-level co-occurrence matrix (GLCM) is a standard method of calculating textural features in 2-D images (Haralick and Shanmugam, 1973). ROI voxel values were scaled from 32-bit to 8-bit integers, and image contrast was enhanced via histogram equalisation for each sample ROI. The 2-D GLCM size scales with the square of the number of image grey levels. A GLCM for an 8-bit image has 2^16^ elements, compared to 2^64^ elements for a 32-bit image, which is on the order of exabytes. Therefore, scaling to 8-bit is customary to give a tractable computation time, 75 min in our case using a Dell PC with 256 GB RAM. Additionally, it was found that a linear scaling of optical CT image intensity to grey-level value suppressed image contrast due to the presence of some highly attenuating artefacts. Histogram equalisation was therefore employed to maintain intra-sample contrast despite sacrificing inter-sample comparability of absolute values. This process can amplify noise, so a median filter with a kernel width of 3 was applied prior to analysis to improve the signal-to-noise ratio (SNR). This width was chosen given the interest in long-range textural features, such as red and white pulp boundaries that change over larger pixel distances.

Standard 2-D GLCMs were calculated for each 2-D *x*–*y* slice in the sample ROIs for a range of pixel displacements and averaged over four angular directions (*θ* = 0°, 45°, 90°, 135°, see Fig. 2) (Haralick and Shanmugam, 1973). However, to take into account the 3-D nature of the data, the GLCM analysis was extended to include a third reference point in the *z* direction, giving rise to a 3-D GLCM (see Fig. 2d.). The 3-D GLCM, *P_d,θ_* for an *n × m × p* image volume, *I,* is defined below (Eq. (2)) where *d* is the pixel displacement and *θ* is the displacement angle, related to pixel position changes ∆*x*, ∆*y* and ∆*z* as follows,

(1) $$\begin{array}{ll} \Delta x=\Delta z=d\cos\theta , & \Delta y=d\sin\theta \end{array}$$

(2) $$P_{d,\theta }\left(i,j,k\right)=\sum_{x=0}^{n-1}\sum_{y=0}^{m-1}\sum_{z=0}^{p-1}\left\{\begin{array}{l} \;\;I\left(x,\;y,\;z\right)=i\\ 1\;,\;\mathrm{if}\;I\left(x+\Delta x,\;y+\Delta y,\;z\right)=j\\ \;\;I\left(x,\;y+\Delta y,\;z+\Delta z\right)=k\\ 0\;,\;\mathrm{otherwise}. \end{array}\right)$$

3-D matrices were calculated for a range of *d* values and averaged over four angular directions (*θ* = 0°, 45°, 90°, 135°). Contrast and homogeneity were calculated from these 3-D GLCMs according to equations adapted from (Chen et al., 2009) (see Eqs. (3) and (4) where *N_g_* is the total number of grey levels). The analysis was not blinded and these endpoints were decided after data acquisition.

(3) $$\mathrm{Contrast}=\sum_{i,j,k=0}^{N_{g}-1}P_{i,j,k}\left[{\left(i-j\right)}^{2}+{\left(i-k\right)}^{2}+{\left(j-k\right)}^{2}\right]$$

(4) $$\mathrm{Homogeneity}=\sum_{i,j,k=0}^{N_{g}-1}\frac{P_{i,j,k}}{1+{\left(i-j\right)}^{2}+{\left(i-k\right)}^{2}+{\left(j-k\right)}^{2}}$$

### Simulation

In order to investigate some of the variables affecting the textural metrics above we performed 2-D GLCM analysis on simple simulations of a single spleen slices, incorporating three different tissue types present within the spleen. At the chosen imaging wavelength of 430 nm, a strong source of optical absorption is haemoglobin in red blood cells (RBCs). The red pulp areas of the spleen transport and trap some RBCs making these areas highly absorbing on our optical CT scans. White pulp areas including lymph nodes do not usually contain RBCs and are therefore far less absorbing. The marginal zone between red and white pulp can trap RBCs to a lesser extent than the red pulp giving it intermediate absorption.

Simulated spleen images were generated incorporating these three tissue types. Each simulated image had four ‘features’ made up of a simulated lymph node and surrounding simulated marginal zone. The lymph nodes were simulated as circles, with constant grey level value of 0. The marginal zone was represented as a circular disc around the lymph node with uniform grey level value of 128 and the surrounding areas simulated the red pulp, with a grey level value of 256. Four simulated spleen images were generated with different lymph node and marginal zone radii, changing the total size of the features and the ratio of marginal zone area within each feature. 2-D contrast was calculated for each case.

## Results

**Fig. 3:**
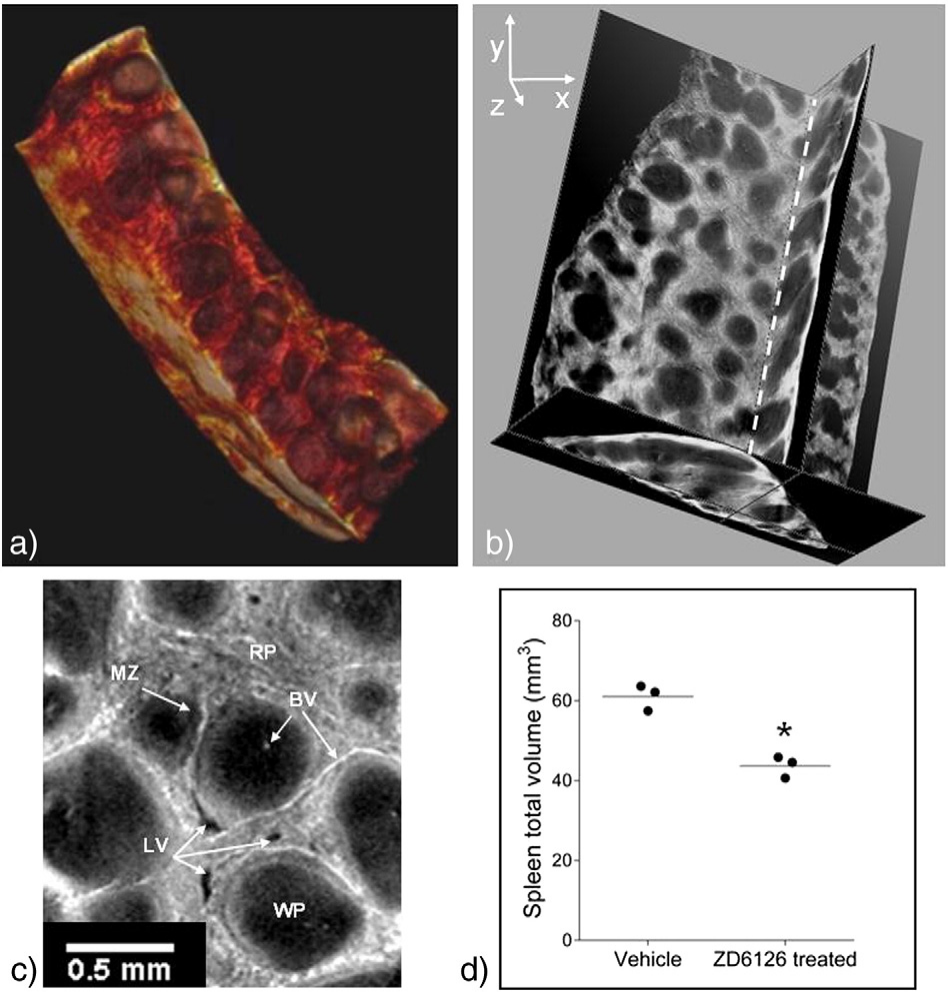
a. A still image from a volume rendering of a ZD6126-treated spleen from Dataset 2 with FOV (5.3 mm)^3^, rendering made using OsiriX software (Rosset et al., 2004). b. A still view of orthogonal slices through a reconstructed volume of another ZD6126-treated spleen with FOV (5.3 mm)^3^. Full representations of these datasets are available online as video clips (see Appendix A). c. A magnified view of a single slice from the volume shown in b., with some anatomical features marked. RP: red pulp, MZ: marginal zone, BV: blood vessel, LV: lymph vessel, WP: white pulp. Note that blood is highly attenuating and appears bright on these scans. d. Total volume of vehicle and ZD6126-treated spleens (lines mark the mean for each group, *p = 0.05).

High contrast data were successfully acquired for each spleen, and all animals were included in analysis. Fig. 3a. and b. display typical examples of the 3-D image datasets, in two complementary representations revealing the classical honeycomb appearance and complex inner structure. Full 3-D representations are available as supplementary video files. Fig. 3c. shows a single 2-D reconstructed slice annotated to show some anatomical features.

The total volume of each spleen is plotted in Fig. 3d. where there is a significant difference between the ZD6126-treated and vehicle groups. Total splenic volumes in the vehicle group ranged from 57.4 mm^3^–63.9 mm^3^, while the ZD6126-treated group volumes ranged from 40.6 mm^3^–45.8 mm^3^ demonstrating a ZD6126-induced median shrinkage of 28% with significance of p = 0.05.

**Fig. 4:**
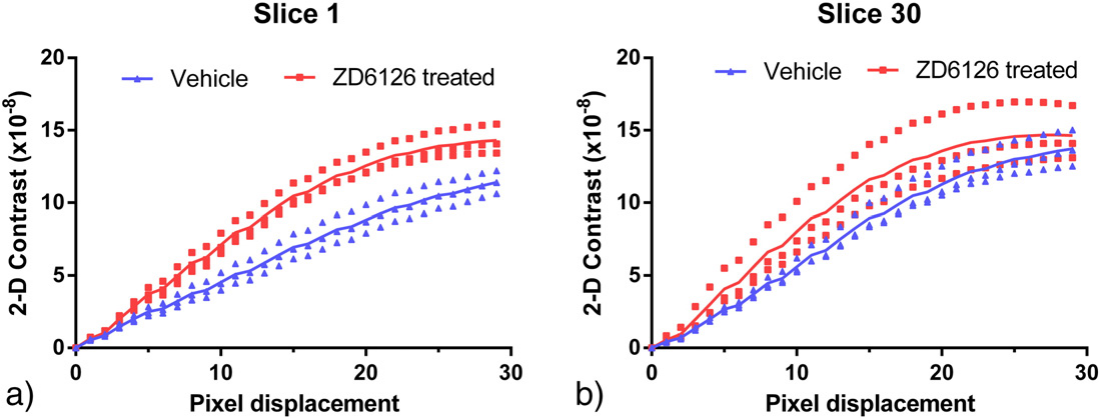
The calculated 2-D contrast parameter for two different 2-D slices from each sample region-of-interest, after histogram equalisation and median filtering, for a range of pixel displacements. The solid lines show the mean values for each group. It is evident that separation of ZD6126-treated and vehicle cohorts depends on the slices compared, the groups are distinct for almost all length scales in slice 1 (a.) but overlap for the majority of pixel displacements in slice 30 (b.).

**Fig. 5:**
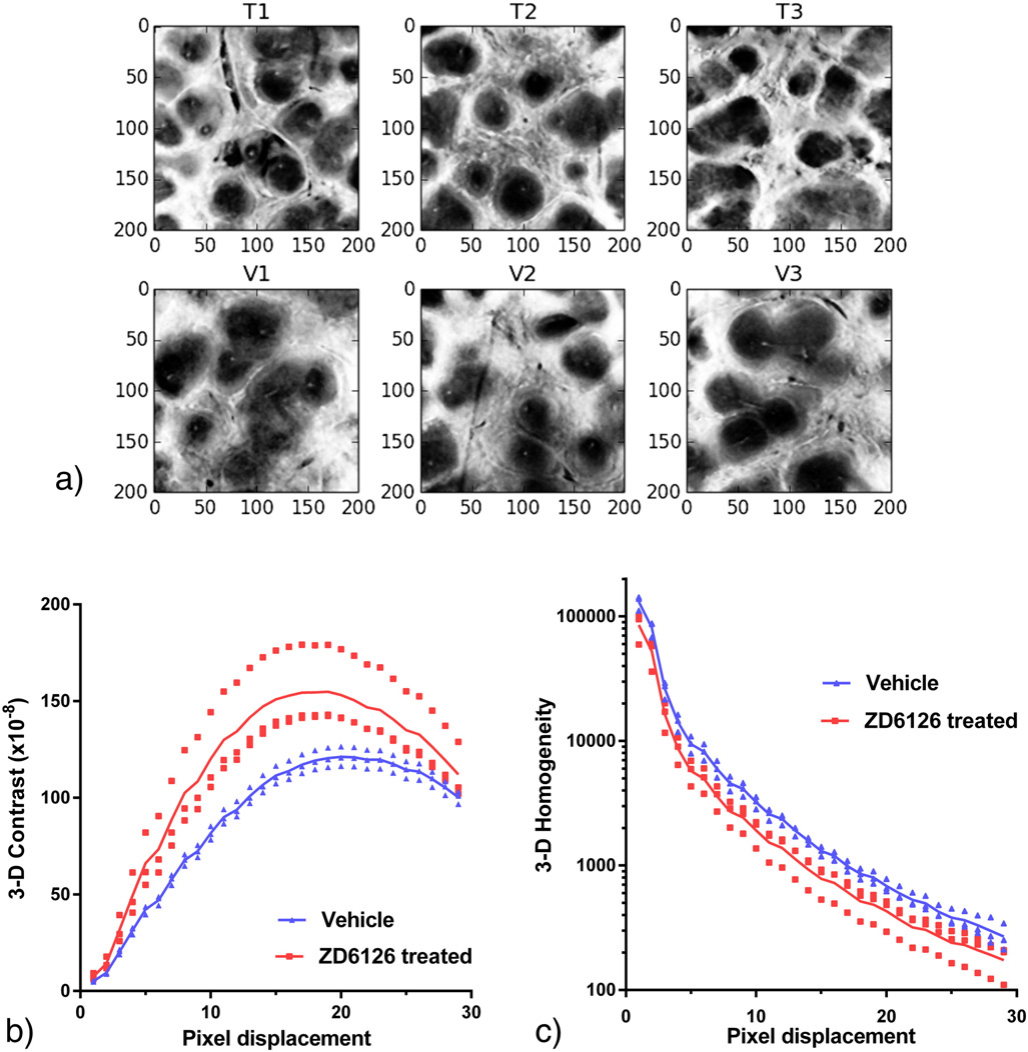
a. Example 2-D slices from each sample region-of-interest after histogram equalisation and median filtering. ZD6126-treated spleens (T1–3) demonstrate visibly sharper boundaries between regions than vehicle spleens (V1–3). b. 3-D contrast, and c. 3-D homogeneity for each sample, for different pixel displacements (average of four angles 0°, 45°, 90° and 135°). The solid lines show the mean values for each group at each pixel displacement and there is a clear separation between the groups for a range of pixel displacements. This gives an indication of the length scales of the features that are different between the groups.

Our initial 2-D GLCM analysis, calculated as specified by (Haralick and Shanmugam, 1973), was found to be slice-dependent (Fig. 4). However, the modified GLCM analysis incorporating three reference points (Eq. (2)) reduced the influence of analysis position on results. Calculation of the 3-D contrast and homogeneity texture parameters defined in Eqs. (3) and (4) showed a clear separation between ZD6126-treated and vehicle groups as shown in Fig. 5b. and c. The position of peak contrast was shifted to smaller pixel displacements for the ZD6126-treated samples with a peak contrast occurring at a distance of 18.2 ± 0.9 pixels for ZD6126-treated samples and 20.2 ± 0.9 pixels for the vehicle cohort, representing a physical difference of 20.8 μm.

**Fig. 6:**
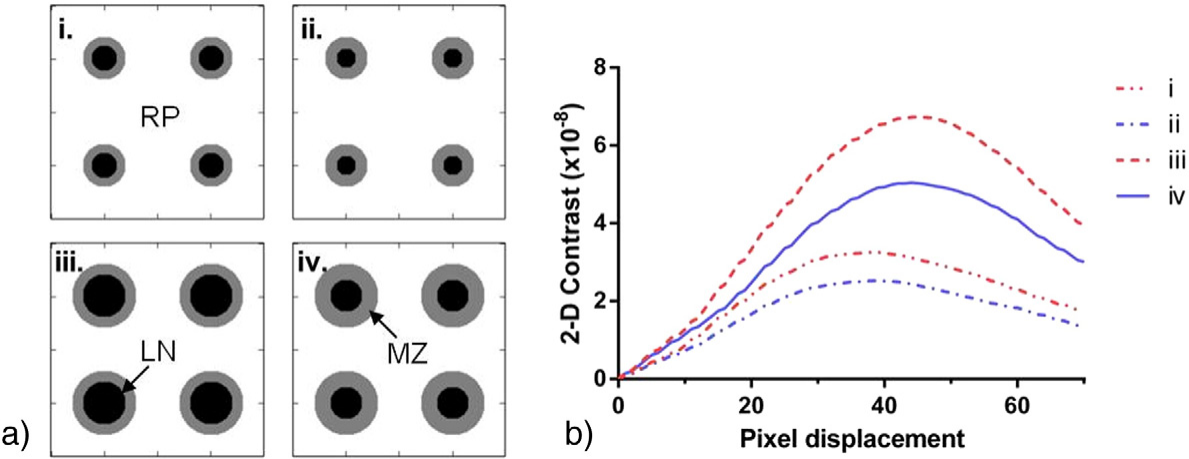
a. Simple simulations of spleen features, RP: red pulp, LN: lymph node, MZ: marginal zone. The overall feature size was varied (i and ii had smaller features than iii and iv), as was the area of MZ in each feature (increased MZ area in samples ii and iv compared to i and iii respectively). b. 2-D contrast for each simulation, i–iv, for a range of pixel displacements. The peak contrast for simulations i and ii was shifted to smaller pixel displacements compared to iii and iv. Decreased MZ area resulted in higher 2-D contrast.

After analysis of 2-D simulations of splenic features it was found that features with smaller radii resulted peak contrast occurring at smaller pixel displacements and simulations with decreased marginal zone area had increased 2-D contrast (Fig. 6).

## Discussion

In this study, we were able to demonstrate the use of endogenous optical CT contrast to provide distinct images of murine spleen microstructure, and its sensitivity to treatment-induced effects on spleen function following administration of the VDA ZD6126. Differences in spleen structure between the two groups were clearly observed via optical CT and changes in total spleen volume, 3-D contrast and homogeneity were determined.

Owing to the distortions induced by physical sectioning of the samples and the other difficulties described in the introduction, assessment of volume is problematic using standard 2-D histology techniques. Thus, the volume difference between ZD6126-treated and vehicle spleens may not have been detected using conventional histology due to the irregularity of spleen shapes. The changes in volume observed in our optical CT images may not be linearly related to actual changes in vivo due to the nature of the clearing process. Dehydration causes tissue shrinkage and the amount of shrinkage may depend on original tissue size and density. Nevertheless, given our observations and the results of previous studies using ZD6126 and another VDA, AVE8062 (Cullis et al., 2006; Guffroy et al., 2004), it is highly likely that the drug has caused the spleen to contract. This is thought to be a consequence of the sensitivity of the atypical sinusoidal, unstable endothelium of the spleen (Groom, 1987) to tubulin-binding VDAs. As shown in Fig. 4, 2-D GLCM statistics gave different results depending on the slice analysed. This is because the complex 3-D structure of the spleen cannot truly be sampled in one slice, emphasising the need for 3-D analysis.

Direct biological interpretation of textural feature statistics is difficult given that many factors contribute to the final result. Qualitatively we noticed that the ZD6126-treated samples appeared to have a steeper gradient of grey values between white and red pulp areas (Fig. 5a.). The results of the simple simulation (Fig. 6) suggest that the higher 3-D contrast of ZD6126-treated spleens at certain pixel displacements could be related to contraction of the marginal zone area leading to apparently sharper definition of the red pulp, supported by the observed difference in volume. This is also consistent with the shift in 3-D contrast peak position to smaller pixel displacement for the ZD6126-treated group, which supports the hypothesis that internal structures are smaller on average in the ZD6126-treated group.

In previous findings, ZD6126 was found to reduce spleen perfusion, associated with red pulp areas as measured by uptake of Hoechst 33342 (Cullis et al., 2006). The reason for the reduction in mean Hoechst perfused area observed may be due to a contraction of the overall marginal zone and red pulp volume. However, this hypothesis is likely to be difficult to test, as the process of staining with Hoechst 33342 involves frozen sections which is currently incompatible with optical CT tissue preparation steps.

## Conclusion

The effects of the VDA ZD6126 on the spleen were detected and characterised in 3-D, using an optical CT anatomical scan sensitive to haemoglobin absorption contrast. In the future this could be extended using optical stains providing additional functional information. For example, India ink would provide detail of perfused vessels and fluorescent micro-particles can indicate the degree of RBC trapping in the red pulp and marginal zones of the spleen. Indeed, there are plenty of data from these initial scans that could be extracted with further computational analysis.

We have shown that spleen imaging using optical CT is potentially a very useful measure of spleen structure in 3-D, complementary to ultra-high resolution 2-D methods such as SEM and TEM. This method can be more straightforward than microvascular-corrosion cast methods, which involve many more preparatory steps prior to imaging and can be susceptible to practical problems in delivering resin down to the capillary level. Further investigation is continuing, including testing different functional stains and characterising the effect of the clearing process on this contractile organ. The promising results of this proof-of-concept study suggest a useful role for optical CT in assessment of drug-induced changes. Future studies with a larger cohort to enable robust statistical testing will establish the significance of the quantitative changes and the sensitivity of the technique.

## Appendix A Supplementary data

Supplementary video 1 volume rendering of a ZD6126-treated spleen image volume from Dataset 2 with FOV (5.3 mm)^3^, rendering made using OsiriX software (Rosset et al., 2004).

Supplementary video 2 orthogonal slices through a reconstructed optical CT image volume of a ZD6126-treated spleen from Dataset 2 with FOV (5.3 mm)^3^.
