# Supplementary figures and images for: Detecting microvascular changes in the mouse spleen using optical computed tomography
Source: Microvasc Res. 2015 Sep;101:96–102. doi: 10.1016/j.mvr.2015.06.008 (PMC4542549; doi:10.1016/j.mvr.2015.06.008)

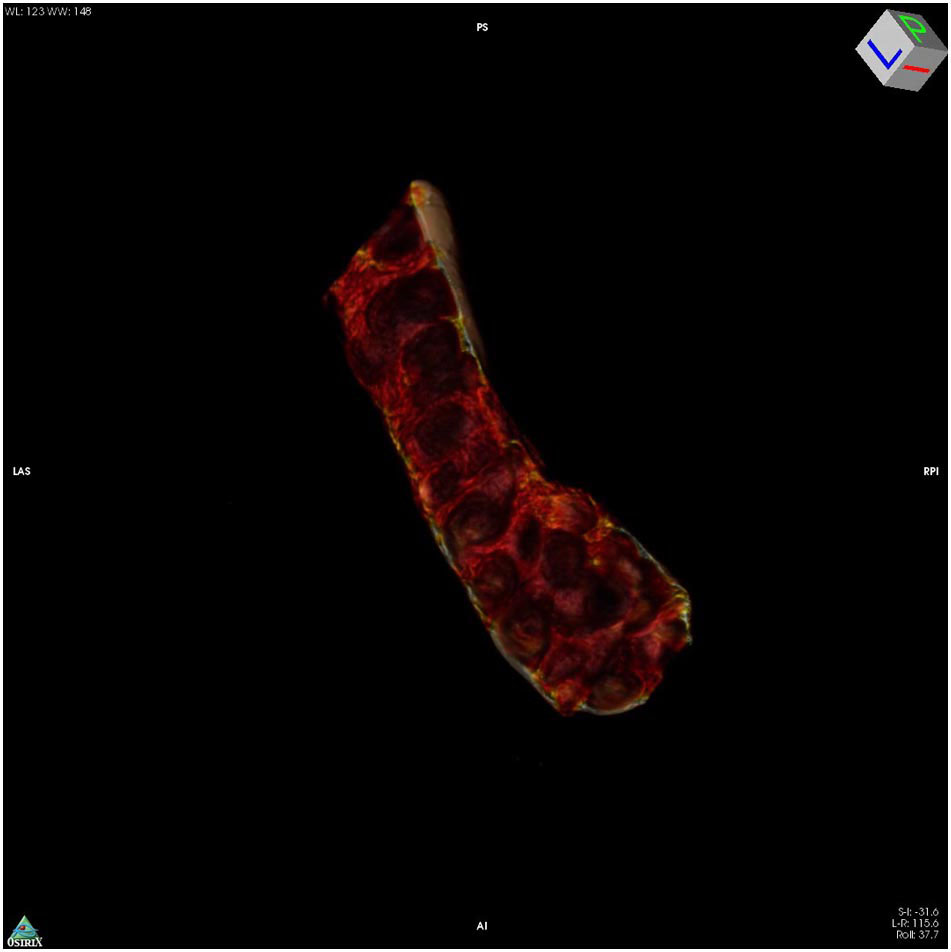

Supplement: Supplementary video 1 — volume rendering of a ZD6126-treated spleen image volume from Dataset 2 with FOV (5.3 mm)3, rendering made using OsiriX software (Rosset et al., 2004). [file mmc1.jpg]

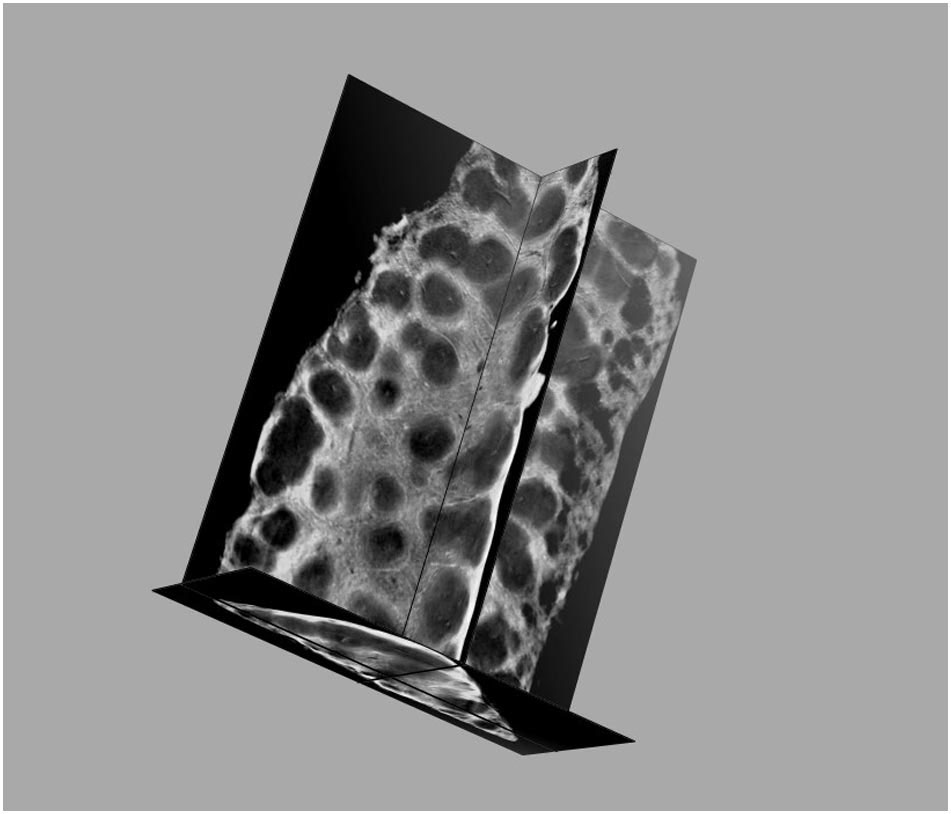

Supplement: Supplementary video 2 — orthogonal slices through a reconstructed optical CT image volume of a ZD6126-treated spleen from Dataset 2 with FOV (5.3 mm)3. [file mmc2.jpg]
